# C‐Terminal Agrin Fragment as a Biomarker for Sarcopenia: A Systematic Review and Meta‐Analysis

**DOI:** 10.1002/jcsm.13707

**Published:** 2025-01-30

**Authors:** Rida Fatima, Yonghoon Kim, Suhyeon Baek, Reema Priyanka Suram, Sun‐Joung Leigh An, Yonggeun Hong

**Affiliations:** ^1^ Department of Rehabilitation Science Graduate School of Inje University Gimhae South Korea; ^2^ Biohealth Products Research Center (BPRC) Inje University Gimhae South Korea; ^3^ Research Center for Aged‐Life Redesign (RCAR) Inje University Gimhae South Korea; ^4^ Department of Occupational Therapy, College of Healthcare Medical Science & Engineering Inje University Gimhae South Korea; ^5^ Department of Physical Therapy, College of Healthcare Medical Science & Engineering Inje University Gimhae South Korea

**Keywords:** C‐terminal agrin fragment, hang grip strength, sarcopenia, skeletal muscle index

## Abstract

**Background:**

Sarcopenia is a gradual decline in skeletal muscle mass and strength, which eventually leads to reduced physical performance. 50% of people aged 60–80 years suffer from sarcopenia. Considering the devastating outcomes and the importance of promoting healthy ageing, the diagnosis and prevention of sarcopenia is of utmost importance. Recently, C‐terminal agrin fragment (CAF) has been identified as an indicator for early diagnosis of sarcopenia. So far, systematic reviews demonstrating CAF as a biomarker for sarcopenia have been conducted, but a meta‐analysis is still needed. This study contains systematic review as well as detailed meta‐analysis to better understand the association of CAF and sarcopenia.

**Methods:**

Articles were primarily obtained from four different databases. Studies demonstrating the association between CAF and sarcopenia were selected. Data extraction and analysis were performed using STATASE 16 software. The risk of bias and quality assessment of each study was carried out using Joanna Briggs Institute (JBI) Critical Appraisal Tool. Meta‐regression, subgroup and sensitivity analysis were conducted to identify the source of heterogeneity.

**Results:**

Seventeen studies were included in the qualitative analysis, out of which 10 were included in the quantitative analysis. The meta‐analysis showed that CAF levels were significantly higher in sarcopenia patients, with an effect size of 1.93 (ROM = 1.93, 95% CI [1.49 to 2.36]; *p* = 0.00) and 1.38 (ROM = 1.38, 95% CI [0.94 to 1.83], *p* = 0.00) when compared with non‐sarcopenic and non‐sarcopenic (other co‐morbidities) group, respectively. CAF levels were also negatively associated with hand grip strength (HGS) and skeletal muscle index (SMI) with an effect size of 1.09 (ROM = 1.09 with 95% CI [1.05 to 1.13], *p* = 0.00) and 1.10 (ROM = 1.10 with 95% CI [1.05 to 1.14], *p* = 0.00), respectively. Meta‐regression and subgroup analysis revealed that although sarcopenia is associated with increasing age, the correlation between CAF and age was statistically insignificant (*p* = 0.44), suggesting that the variation of age among sarcopenia patients could be source of heterogeneity among studies. All the studies included in the meta‐analysis reported low risk of bias.

**Conclusions:**

Our meta‐analysis concluded that elevated CAF levels were associated with sarcopenia and decreased HGS and SMI. CAF could serve as a valuable marker for the early detection and monitoring of sarcopenia, ultimately facilitating the management and treatment of this debilitating condition.

## 1 Introduction

Sarcopenia, as defined by European Working Group on Sarcopenia in Older People (EWGSOP), is a progressive decline in muscle mass and strength, leading to reduced physical performance [1]. Its prevalence escalates with age, affecting 5%–13% of individuals aged 60–70 and 11%–50% of those aged 80 and above [2]. Beyond its impact on the elderly, sarcopenia significantly affect patients with long‐term care or multiple morbidities, with 30%–50% experiencing secondary sarcopenia [3]. Considering the devastating outcomes and to promote healthy ageing, early diagnosis and intervention are crucial to mitigate its debilitating effects [4, 5, 6]. Up to the date, researchers have conducted systematic reviews to understand the biology between CAF and sarcopenia; however, the need for a comprehensive meta‐analysis is still there. To eliminate this need, the current study aims to provide a detailed meta‐analysis along with systematic review.

Currently, various clinical methods are used to assess sarcopenia. Muscle mass is typically measured using magnetic resonance imaging (MRI), dual‐energy X‐ray absorptiometry (DEXA), bioelectrical Impedance analysis (BIA) and computed tomography (CT) [7, 8]. Muscle strength and physical performance are assessed using questionnaires such as the SARC‐F (strength, assistance with walking, rising from a chair, climbing stairs and falls) or objective tools like the Short Physical Performance Battery (SPPB) test [9]. Muscle mass measuring technologies are expensive and not widely available in community care settings [7, 10]. Furthermore, the validity of data from questionnaires and performance‐based tools can be compromised by cognitive or psychological issues, leading to inaccurate results [10]. Therefore, a reliable method for assessing sarcopenia that overcomes these limitations is needed. One promising approach is the detection of specific biomarkers in blood or plasma [11].

Understanding the pathophysiology of sarcopenia is essential for identifying specific biomarkers [12]. A key factor in the development and progression of sarcopenia is age‐related alterations in the neuromuscular junction (NMJ) due to decreased reinnervation [13]. As people age, significant remodelling occurs in the NMJ to maintain nerve‐muscle communication. Agrin, a heparan sulfate proteoglycan, plays a crucial role in forming and stabilizing postsynaptic structures of NMJs [14]. During the remodelling process, the proteolytic cleavage of neuro‐trypsin (a neuronal protease) oxidizes agrin into 22‐kDa C‐terminal agrin fragment (CAF), which can be easily detected in blood serum [15]. The destruction of agrin inactivates the cholinergic receptors in the presynaptic membrane leading to the accumulation of denervated muscle fibres [16]. This process results in gradual loss of muscle mass accompanied by decreased muscle strength, a characteristic of sarcopenia. Hence, CAF can be considered an early biomarker for diagnosing sarcopenia [17].

Recent studies have explored the association between CAF levels and sarcopenia, suggesting CAF as a potential early diagnostic biomarker. For instance, in a study evaluating primary and secondary sarcopenia patients, CAF levels were significantly associated with muscle mass, hand grip strength (HGS) and physical performance [18]. Drey et al. [19] found that reduced appendicular lean mass was linked to increased CAF concentration, potentially leading to development of sarcopenia in community‐dwelling older adults. Kalinkovich et al. [20] identified age‐related NMJ dismantling and increased CAF production as factors elevating sarcopenia onset and progression. Pranathi et al. [21] observed higher serum CAF concentrations in type 2 diabetes (T2D) patients compared with prediabetics (PD) and healthy individuals, proposing CAF as a biomarker for evaluating sarcopenia in T2D patients.

There is substantial evidence supporting various biomarkers for sarcopenia assessment [22]. Recently, CAF has gained particular importance for diagnosing early‐onset of sarcopenia [17, 18, 19]. Studies have suggested that CAF has the potential to identify early denervation at NMJs and can serve as a valid diagnostic tool for muscle wasting and atrophy associated with sarcopenia [11]. Therefore, this review aims to explore and establish a comprehensive understanding of the association between CAF and both primary and secondary sarcopenia, thereby validating CAF as an emerging biomarker for sarcopenia.

## 2 Methods

The Preferred Reporting Items for Systematic Reviews and Meta‐Analysis (PRISMA) guidelines [23] were followed for this systematic review and meta‐analysis. Ethical considerations were addressed in accordance with the guidelines of Wager et al. [24] The study protocol was registered in 2024 with the ‘International Prospective Register of Systematic Reviews’ (PROSPERO) (https://www.crd.york.ac.uk/PROSPERO/) under the registration number: CRD42024551517.

### 2.1 Search Strategy

A comprehensive search was conducted in four electronic databases: PubMed, EMBASE, ISI Web of Science and Cochrane Library. The originally published articles included in this review that reported data for qualitative and quantitative analysis were found from 2014 to 2024. The keywords used in the search included ‘sarcopenia’, ‘muscle wasting’, ‘muscle atrophy’ and ‘C‐terminal agrin fragment’. Additionally, forward and backward citation searches for each selected article were done in Google Scholar to identify more relevant articles.

After retrieving all the studies from the electronic databases (PubMed, EMBASE, ISI Web of Science and Cochrane Library), duplicates were removed using ENDNOTE 20 software. Each article was then screened individually using the Rayyan Intelligent Systematic Review tool. Articles that contained only reviews or abstracts were excluded manually [25].

The potentially eligible articles for the meta‐analysis were retrieved for primary and secondary assessment. Two independent authors were assigned to analyze the titles and abstracts and assess their relevance to the current study. Any conflicts or disagreements regarding study selection were resolved through consultation with a third author. Finally, the full texts of the remaining articles were obtained and extensively screened to determine which articles would be included in the systematic review and meta‐analysis.

### 2.2 Inclusion and Exclusion Criteria

Based on the PCC format, studies were included if they met the following criteria:

- Population: Individuals > 50 years of age.
- Concept: CAF concentrations in blood (plasma or serum).
- Context: Sarcopenic and non‐sarcopenic individuals.

Studies were excluded if they:

- Lacked a sarcopenic population.
- Did not report CAF concentrations.
- Presented data in a format other than mean ± SD or mean ± SE for CAF levels.
- Involved animal studies.

### 2.3 Data Extraction

The data sets obtained after final screening were analyzed independently by two authors. Any conflicts or disagreements regarding inclusion/exclusion criteria were resolved by a third author. The data extracted from the selected studies included the following: first author, country and time of publication, study design, number and age of sarcopenic and non‐sarcopenic patients, sarcopenia assessment criteria, mean (SD) of CAF levels in sarcopenic and non‐sarcopenic group and associated factors of sarcopenia. Data presented only in graphs and figures were extracted using GetData Graph Digitizer (https://getdata‐graph‐digitizer.software.informer.com/) software. For data reported as mean and standard error (SE), SE was converted to standard deviation (SD) using the formula: SD = SE * √sample size [26].

### 2.4 Risk of Bias/Quality Evaluation

Two independent authors were assigned to perform the quality assessment and evaluate the risk of bias using the Joanna Briggs Institute (JBI) Critical Appraisal Tool for cross‐sectional [27], cohort [28] and case–control studies [29]. Any conflicts or disagreements were resolved by a third author. JBI critical appraisal tool consists of 8 assessment questions for cross‐sectional studies, 10 for case‐control studies, and 11 for cohort studies, each containing 1 point. Each question consists of four possible answers: ‘yes’, ‘no’, ‘unclear’ and ‘not applicable’. Scoring was based on the total number of ‘yes’ answers for each study.

Studies were evaluated based on following criteria:

- Studies with more than 70% ‘yes’ answers were considered to have low risk of bias.
- Studies with 50 to 69% ‘yes’ answers were considered to have a moderate risk of bias.
- Studies with less than 49% ‘yes’ answers were considered to have a high risk of bias [30].

### 2.5 Statistical Analysis

For conducting the meta‐analysis, the ratio of mean (ROM) was calculated to compare the sarcopenic and non‐sarcopenic groups [31]. Because the units of CAF concentration varied across studies, we unified all the units by obtaining the ROM of CAF concentration from each study as a measurement of effect size, with a 95% confidence interval [32, 33]. The formula used for calculating ROM was $\mathrm{ROM}=\frac{\text{mean}\;\mathrm{CAF}\;\text{concentration of sarcopenic group}}{\text{mean}\;\mathrm{CAF}\;\text{concentration of non‐sarcopenic group}}$. ROM greater than 1 indicates that the CAF concentration in the sarcopenic group is higher than in the non‐sarcopenic group, whereas an ROM less than 1 indicates that the CAF concentration is higher in the non‐sarcopenic group [34]. The Taylor series approach was used to calculate the pooled standard error (SEPool) [35]. Heterogeneity was assessed using the *I* ^2^ test in STATASE 16 (Stata Corp., College Station, TX, USA) software [36] employing the generic inverse variance with a random‐effects model and the restricted maximum likelihood method. An *I* ^2^ > 50% is indicative of considerable heterogeneity.

To determine the cause of heterogeneity, we performed subgroup analysis, meta‐regression and leave‐one‐out sensitivity analysis. Publication bias was assessed using Egger's test and funnel plot assessment in Stata SE 16 software [36, 37, 38].

## 3 Results

### 3.1 Study Selection

**FIGURE 1:**
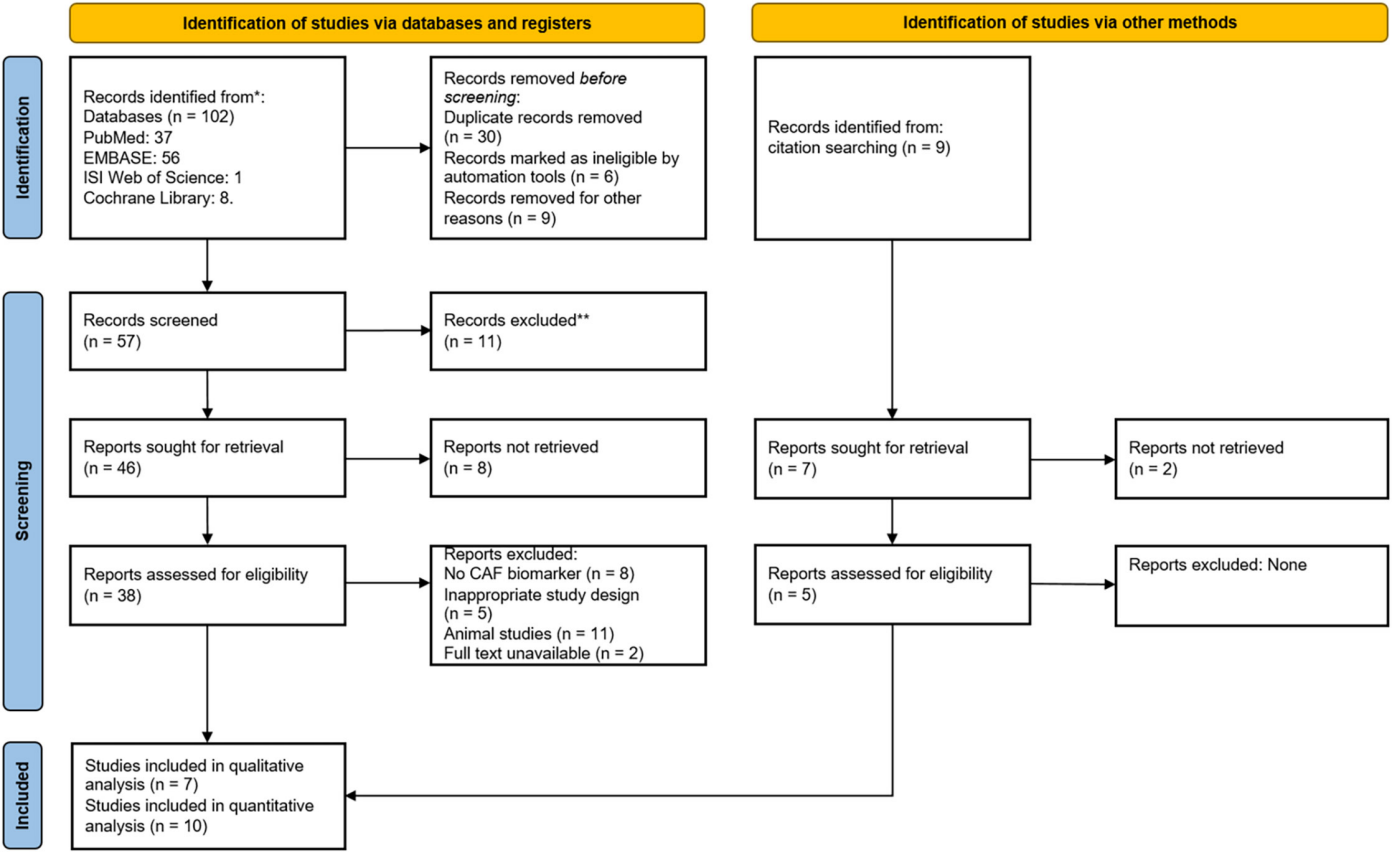
PRISMA flow diagram for eligible studies.

A comprehensive literature search yielded 102 articles from PubMed, EMBASE, ISI Web of Science and Cochrane Library, as shown in Figure 1. After removing duplicates and ineligible studies, 57 studies were evaluated based on their titles and abstracts, resulting in the exclusion of 11 studies. The remaining 38 studies underwent full‐text assessment. Additionally, a manual search, including forward and backward citation, yielded nine studies, of which two were excluded based on ineligibility.

Finally, after conducting a full‐text assessment of 38 studies, 17 studies [21, 39, 40, 41, 42, 43, 44] were included in the systematic review, out of which 10 studies [10, 11, 17, 45, 46, 47, 48, 49, 50, 51] were included in the meta‐analysis.

The PRISMA flow diagram (Figure 1) visually represents the study selection process.

### 3.2 Assessment of Quality and Risk of Bias

As shown in Tables S1–S3, all the included studies were rated as having ‘low risk of bias’ as measured by the JBI critical appraisal tool for cross‐sectional, cohort and case–control studies, respectively. Data were extracted from these studies and included in the meta‐analysis. None of the studies were rated as having a ‘high risk of bias’. Although the JBI tool is a valuable assessment tool, it is important to acknowledge that it may not capture all potential biases.

### 3.3 Characteristics of Included Studies

Table 1 presents the characteristics of the 17 studies included in this review. All studies were published between 2014 and 2024 and included cross‐sectional, cohort and case–control designs. In total, there were 1837 sarcopenia individuals and 1553 non‐sarcopenic individuals.

**TABLE 1:** Characteristics of eligible studies.

Study	Study design	Sample	Method for obtaining sample	Participant (*n*)	Non‐sarcopenia		Sarcopenia		Sarcopenia assessment criteria	CAF levels (mean ± SD)	Associated factors of sarcopenia
					No. of (*n*)	Age of (*n*) (mean ± SD) (years)	No. of (*n*)	Age of (*n*) (mean ± SD) (years)			
Marzetti et. al, 2014 (Italy)	Cross‐sectional	Serum	Commercial ELISA (NTCAF ELISA, Neurotune, Schlieren‐Zurich, Switzerland)	Hip fracture patients	F = 29, M = 6	83.6 ± 8.7	F = 3, M = 4	84.1 ± 9.1	EWGSOP	Sarcopenia (172.2 ± 47.35), non‐sarcopenia (93 ± 43.77)	Elevated CAF levels were associated with lower SMI and HGS.
Landi et. al, 2016 (Italy)	Cohort	Serum	Commercial ELISA (NTCAF ELISA, Neurotune, Schlieren‐Zurich, Switzerland)	Multimorbid patients	F = 155, M = 76	85.1 ± 4.3	F = 70, M = 31	87.5 ± 5.4	EWGSOP	Sarcopenia (96.99 ± 5.4), non‐sarcopenia (76.54 ± 2.15)	Elevated CAF levels were associated with lower muscle mass, gait speed and HGS.
Asima Karim et. al, 2022 (UAE)	Cohort	Plasma	ELISA (NTCAF ELISA, Neurotune, Schlieren‐Zurich, Switzerland)	Parkinson patients	Total = 73 (sex not specified)	68.3 ± 6.4	Total = 69 (sex not specified)	71.2 ± 6.5	EWGSOP	Sarcopenia (243.2 ± 33), non‐sarcopenia (131.9 ± 37.1)	Elevated CAF levels were associated with lower SMI, HGS and gait speed.
Asima Karim et. al, 2021 (UAE)	Cohort	Plasma	ELISA (NTCAF ELISA, Neurotune, Schlieren‐Zurich, Switzerland)	COPD patients	F = 0, M = 84	67.9 ± 5.5	F = 0, M = 77	69.3 ± 6.2	EWGSOP	Sarcopenia (224.3 ± 49.6), non‐sarcopenia (135.6 ± 28.7)	Elevated CAF levels were associated with lower SMI, HGS and gait speed.
Castellano et. al, 2019 (Spain)	Cross‐sectional	Plasma	Commercial ELISA (NTCAF ELISA, Neurotune, Schlieren‐Zurich, Switzerland)	Hip fracture patients	F = 6, M = 3	86.2 ± 6	F = 111, M = 29	87.8 ± 4.8	EWGSOP	Sarcopenia (531.1 ± 1054.1), non‐sarcopenia (440.6 ± 632.4)	Not specified
Jedd Pratt et. al, 2021 (Ireland)	Cohort	Plasma	Commercial ELISA (Abcam #ab216945)	Community dwelling older adults	269 (sex not specified)	N/A	31 (sex not specified)	N/A	EWGSOP2	Sarcopenia (3.13 ± 1.33), non‐sarcopenia (2.65 ± 0.49)	Elevated CAF levels were associated with lower grip strength
Hettwer et. al, 2013 (Switzerland)	Case–control	Serum	Western Blot	Community dwelling older adults	F = 28, M = 32	71.2 ± 5.6	F = 34, M = 39	70.8 ± 5.5	Sarcopenia	Sarcopenia (4.71 ± 2.6), non‐sarcopenia (2.64 ± 0.97)	Elevated CAF levels were associated with lower grip strength
Qun Xu et. al, 2023 (China)	Cross‐sectional	Serum	ELISA (MyBioSource)	Community dwelling older adults	F = 41, M = 48	79.03 ± 10.030	F = 15, M = 23	83.05 ± 7.389	AWGS 2019	Sarcopenia (729.01 ± 194.519), non‐sarcopenia (681.86 ± 198.133)	Elevated CAF levels were associated with lower grip strength
Al Rubaye et. al, 2020 (Iraq)	Case–control	Serum	Automated ALISA (ELisys Uno, human product)	Community dwelling older adults	F = 30, M = 30	67.98 ± 6.90	F = 55, M = 55	66.41 ± 7.53	Sarcopenia	Sarcopenia (8.2 ± 6.05), non‐sarcopenia (3.5 ± 3.91)	Not specified
Asima Karim et. al, 2022 (UAE)	Cohort	Plasma	ELISA	COVID patients	F = 0, M = 62	N/A	F = 0, M = 25	N/A	EWGSOP2	Sarcopenia (217 ± 39.3), non‐sarcopenia (72.3 ± 32.3)	Elevated CAF levels were associated with lower HGS, gait speed and SMI
Kamiya et. al, 2023 (Japan)	Cohort	Serum	Commercial ELISA (Glory Science Co., Ltd., Shanghai, China)	Community dwelling older women	—	—	F = 515, M = 0	74.3 ± 6.3	Not specified	—	Elevated CAF levels were associated with lower muscle mass
Qaisar et. al, 2021 (Sharjah)	Cohort	Serum	ELISA (NTCAF ELISA, Neurotune, Schlieren‐Zurich, Switzerland)	CHF and COPD patients	87 (sex not specified)	62.6 ± 5.5	COPD (*n*) = 86, CHF (*n*) = 81 (sex not specified)	COPD= 64.3 ± 3.7, CHF = 66.9 ± 5.4	SPPB Score	—	Elevated CAF levels were associated with lower SMI, HGS and gait speed
Racha et. al, 2022 (India)	Case–control	Serum	ELISA (FineTest, Wuhan Fine Biotech Co., Ltd. China, Cat. No. EH4820)	Type 2 diabetes mellitus patients	24 (sex not specified)	33.9 ± 11.2	42 (sex not specified)	48.5 ± 6.7	Not specified	—	Higher CAF levels were associated with sarcopenia, low muscle mass and strength
Scherbakov et. al, 2015 (Germany)	Cohort	Serum	ELISA (NTCAF ELISA, Neurotune, Schlieren‐Zurich, Switzerland)	Stroke patients	F = 17, M = 9	67 ± 8	F = 49, M = 74	70 ± 11	Not specified	—	Higher CAF22 levels were associated with low muscle mass and physical performance
Qaisar et. al, 2020 (Sharjah)	Cohort	Plasma	ELISA (NTCAF ELISA, Neurotune, Schlieren‐Zurich, Switzerland)	COPD, Asthma and Pulmonary TB	F = 0, M = 101	65.1 ± 5.5	COPD (*n*) F = 0, M==100, Asthma (*n*) F = 0, M = 87, Pulmonary TB (*n*) F = 0, M = 83	COPD = 68.1 ± 6.5, Asthma = 58.7 ± 4.2, Pulmonary TB = 62.8 ± 5.1	Not specified	—	Higher CAF22 levels were associated with low muscle strength and physical performance
Steinbeck et. al, 2015 (Germany)	Cohort	Serum	ELISA (NTCAF ELISA, Neurotune, Schlieren‐Zurich, Switzerland)	CHF patients	—	—	—	—	—	—	Elevated CAF22 levels were associated with muscle wasting.
Jedd Pratt et. al, 2024 (UK)	Cross‐sectional	Plasma	ELISA (#ab216945, Abcam, Cambridge, UK)	Community dwelling older adults	343(sex not specified)	—	49 (sex not specified)	—	EWGSOP2	Sarcopenia (3115.1 ± 716.8), non‐sarcopenia (2522.7 ± 672.28)	Elevated CAF levels were associated with lower HGS.

Abbreviations: COPD, chronic obstructive pulmonary disease; COVID, coronavirus disease 2019; CHF, congestive heart failure; TB, tuberculosis; EWGSOP, European Working Group on Sarcopenia in Older People; AWGS, Asian Working Group of Sarcopenia; SPPB, short physical performance battery; CAF, C‐terminal agrin fragment; HGS, hand grip strength; SMI, skeletal muscle index.

The studies were conducted in following countries: Italy, UAE, Spain, Ireland, Switzerland, China, Iraq, Japan, Sharjah, India, Germany and the United Kingdom. The CAF samples were obtained from serum and plasma. Samples were categorized into sarcopenia group (individuals with primary or secondary sarcopenia) and non‐sarcopenia group (individuals with other co‐morbidities). The age of sarcopenia patients included in the studies ranged from 48.5 ± 6.7 to 87.6 ± 4.9 years.

### 3.4 Meta‐Analysis

### 3.5 CAF Levels in Sarcopenic and Non‐Sarcopenic Group

Out of 10 studies included in this meta‐analysis, seven studies reported data about the difference of CAF levels between sarcopenic and non‐sarcopenic individuals. Out of seven studies, four studies [11, 17, 50, 51] included individuals with primary sarcopenia in the sarcopenic group, whereas the remaining three studies [47, 48, 49] included individuals with secondary sarcopenia in the sarcopenic group.

**FIGURE 2:**
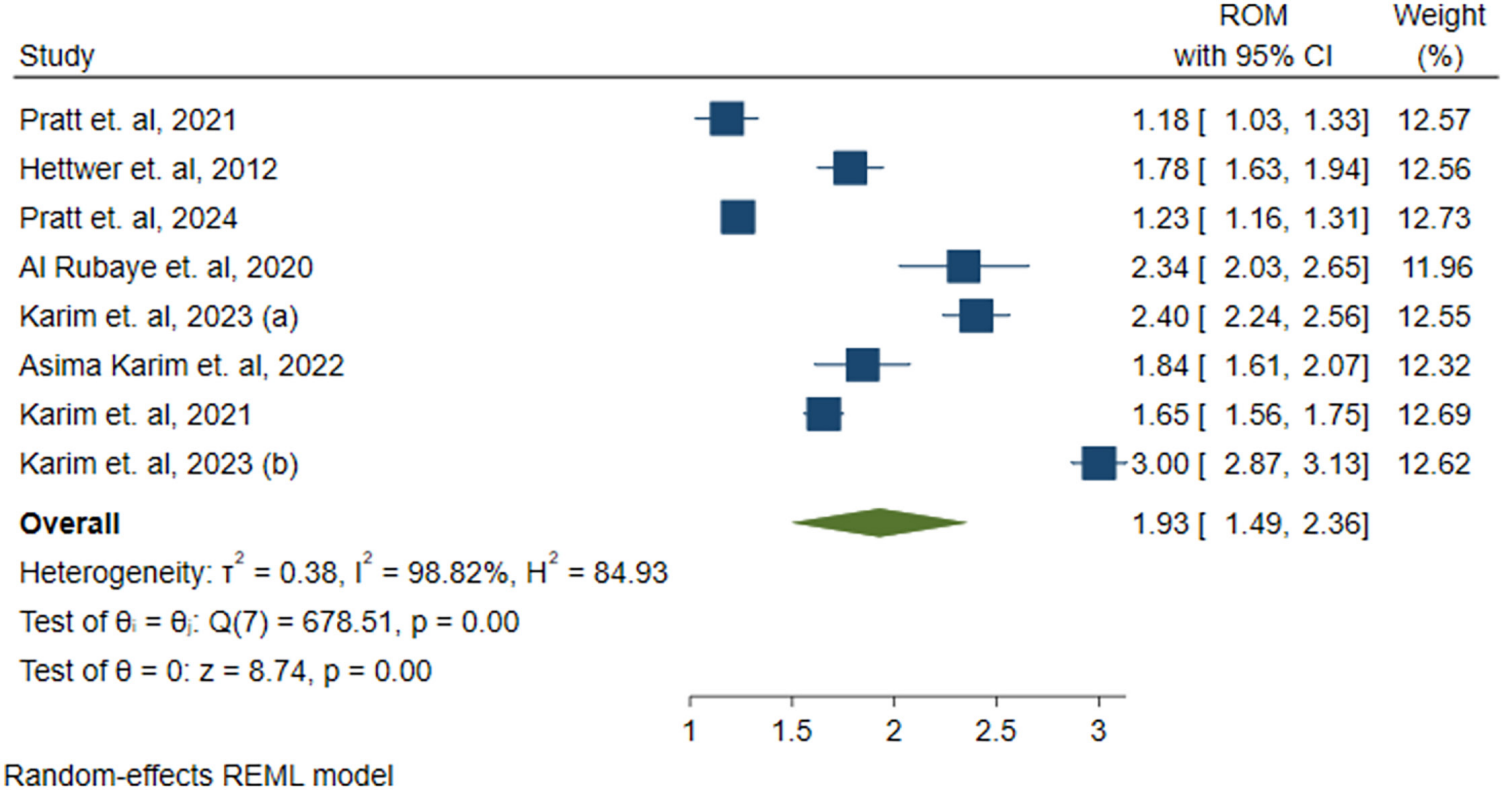
CAF concentration in sarcopenic individuals compared with non‐sarcopenic individuals. Description Forest plot representing ratio of mean (ROM) of C‐terminal agrin fragment (CAF) levels in sarcopenic and non‐sarcopenic group. ROM with their corresponding 95% confidence intervals (CI) of each study are indicated by blue coloured squares. The size of square represents the weight of each study in random‐effect method of meta‐analysis. The diamond shape indicates the summary estimate of ROM with its 95% CI.

In all the studies, CAF levels were significantly higher in the sarcopenic group, with an effect size of 1.93 (ROM = 1.93, 95% CI [1.49 to 2.36]; *p* = 0.00) compared with non‐sarcopenic group, as shown in Figure 2. Given the considerable heterogeneity among studies (Figure 2: *I* ^2^ = 98.82%, *p* = 0.00), we performed a meta‐analysis using the generic inverse variance with random‐effects and restricted maximum likelihood method.

Publication bias was assessed using a funnel plot (Figure S1) and Egger's regression test (*p* = 0.37). The symmetrical funnel plot and nonsignificant Egger's regression test indicated that the studies did not contain a risk of publication bias.

### 3.6 CAF Levels in Sarcopenic and Non‐Sarcopenic (Individuals With Co‐Morbidities Other Than Sarcopenia) Individuals

**FIGURE 3:**
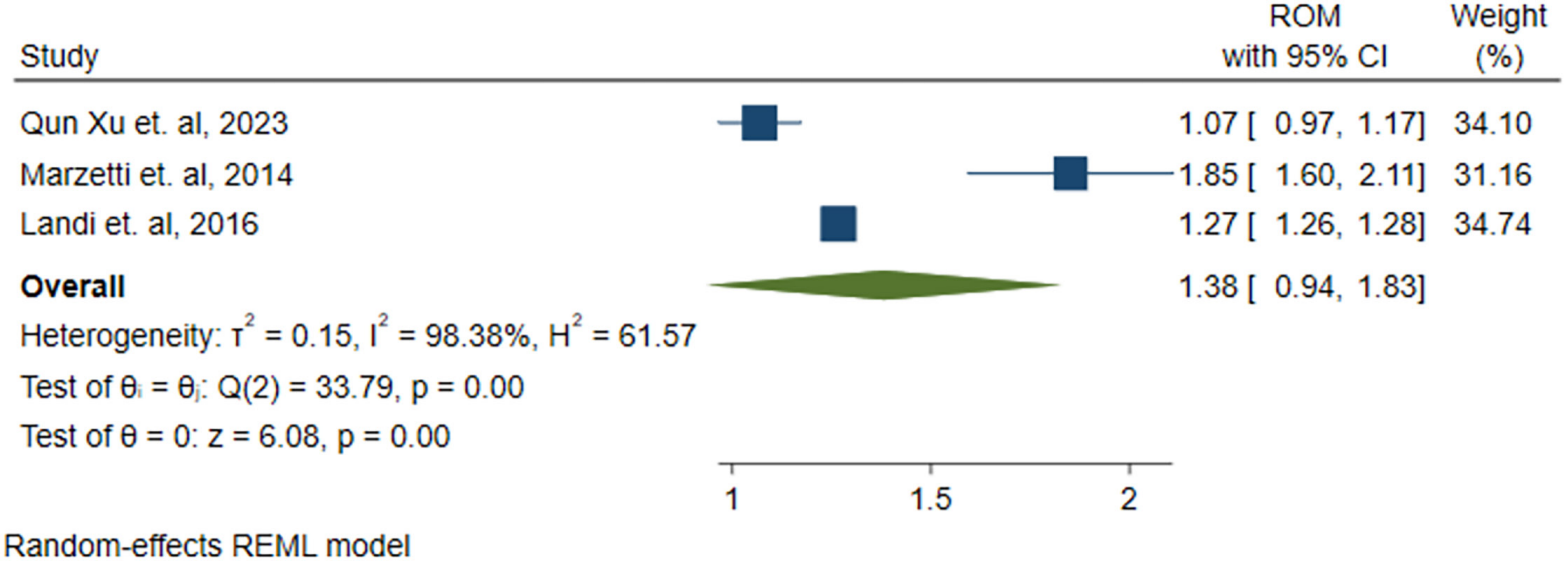
CAF concentration in sarcopenic individuals compared with non‐sarcopenic individuals (individuals with co‐morbidities other than sarcopenia). Description: Forest plot representing ratio of mean (ROM) of C‐terminal agrin fragment (CAF) levels in sarcopenic and non‐sarcopenic group. ROM with their corresponding 95% confidence intervals (CI) of each study are indicated by blue coloured squares. The size of square represents the weight of each study in random‐effect method of meta‐analysis. The diamond shape indicates the summary estimate of ROM with its 95% CI.

The remaining three studies [10, 46, 50] out of the 10 included in our meta‐analysis reported data for CAF levels in sarcopenia group compared with non‐sarcopenia group (containing individuals with co‐morbidities other than sarcopenia). In all three studies, the sarcopenic groups consisted of individuals with secondary sarcopenia. This meta‐analysis also demonstrated that participants in the sarcopenic group had significantly increased levels of CAF, with an effect size of 1.38 (ROM = 1.38, 95% CI [0.94 to 1.83], *p* = 0.00) compared with the non‐sarcopenic group, as shown in Figure 3.

The considerable heterogeneity (Figure 3: *I* ^2^ = 98.38%, *p* = 0.00) was assessed using the generic inverse variance with random‐effects and restricted maximum likelihood method of meta‐analysis. To check for publication bias, we only performed Egger's regression test (*p* = 0.19) and found no significant risk of publication bias. Substantial heterogeneity was observed (*I* ^2^ = 98.38%, *p* < 0.001), leading to the use of a random‐effects model. Egger's test did not indicate significant publication bias (*p* = 0.19).

### 3.7 Association of CAF Levels With HGS and Skeletal Muscle Index (SMI)

HGS and SMI are primary tools for assessing sarcopenia [52]. The EWGSOP defines low muscle strength as HGS < 30 kg and < 20 kg and SMI as < 7 kg/m^2^ and < 5.5 kg/m^2^ in males and females, respectively [52]. To further validate CAF as a biomarker for sarcopenia, we performed a meta‐analysis to examine the association of CAF concentration with HGS and SMI.

**FIGURE 4:**
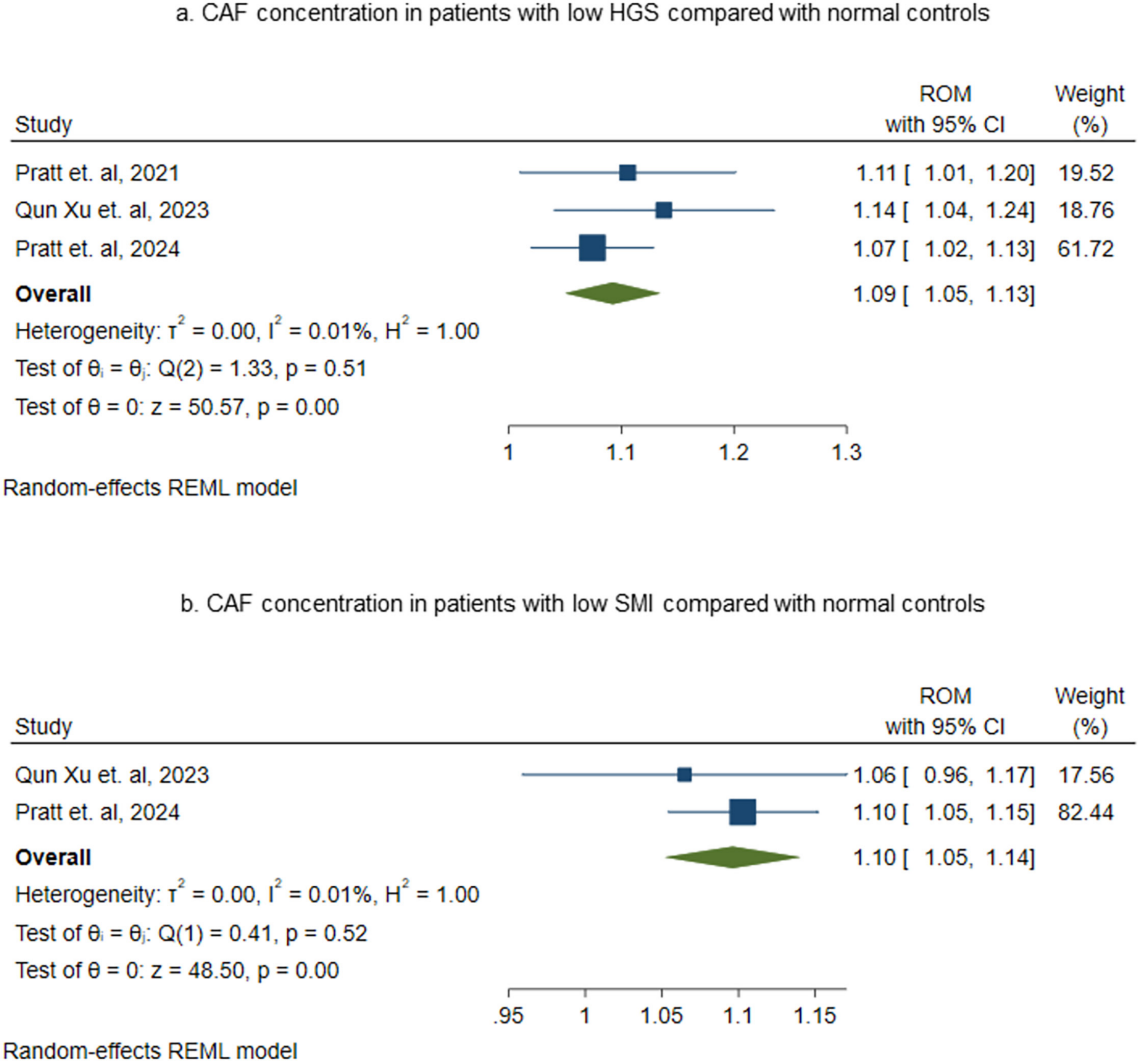
CAF concentration in individuals with low HGS and low SMI compared with normal HGS and SMI individuals. Description: (a) Forest plot representing ratio of mean (ROM) of C‐terminal agrin fragment (CAF) levels in individuals with low HGS compared with individuals having normal HGS. ROM with their corresponding 95% confidence intervals (CI) of each study are indicated by blue coloured squares. The size of square represents the weight of each study in random‐effect method of meta‐analysis. The diamond shape indicates the summary estimate of ROM with its 95% CI. (b) Forest plot representing ratio of mean (ROM) of C‐terminal agrin fragment (CAF) levels in individuals with low SMI compared with individuals having normal SMI. ROM with their corresponding 95% confidence intervals (CI) of each study are indicated by blue coloured squares. The size of square represents the weight of each study in random‐effect method of meta‐analysis. The diamond shape indicates the summary estimate of ROM with its 95% CI.

In Figure 4a, individuals in the sarcopenic group had lower HGS compared with those in the non‐sarcopenic group, who had normal HGS. Individuals with lower HGS had significantly higher levels of CAF concentration, with an effect size of 1.09 (ROM = 1.09 with 95% CI [1.05 to 1.13], *p* = 0.00) compared with individuals who had normal HGS. Figure 4b shows increased CAF concentration in individuals with lower SMI compared with those of normal SMI individuals, with an effect size of 1.10 (ROM = 1.10 with 95% CI [1.05 to 1.14], *p* = 0.00).

Because the cut‐off values for muscle mass and strength in males and females are significantly different, a meta‐analysis stratifying the subjects by their sex is required. Due to lack of enough studies reporting data regarding CAF levels in males and females, the current review could not include meta‐analysis based on sex of study participants. Therefore, further studies are needed to address this research gap.

### 3.8 Subgroup, Meta‐Regression and Sensitivity Analysis

To find out the source of heterogeneity, we conducted a subgroup analysis for age, the source of CAF (serum and plasma), variations in sarcopenia group and assessment criteria of sarcopenia (Figures S2–S5).

As shown in Figure S2, the age subgroup analysis revealed that CAF concentration was higher among sarcopenic individuals with age between 50–60 years (ROM = 2.21 with 95% CI [1.19 to 3.23], *p* = 0.00), while individuals aged 60–70 years had lower CAF levels (ROM = 1.71 with 95% CI [1.06 to 2.36], *p* = 0.00). The heterogeneity in these groups was high (*I* ^2^ = 99.56% and 97.92%, respectively). However, the age group 70–80 years showed nonsignificant results (*p* = 0.68) with no heterogeneity (*I* ^2^ = 0.00%), indicating that the age variation among sarcopenic individuals might contribute to the overall heterogeneity. Around 50–60 years of age, the rapid muscular ageing process leads to an increase in neuromuscular instability which significantly produces higher CAF, allowing the neuromuscular system to cope with early muscle loss. As the age progresses to 60–70 years, the muscle atrophy becomes more pronounced and thus diminishes the neuromuscular instability, reducing the production of CAF [4].

Meta‐regression analysis was carried out to find the correlation between CAF concentration and the age of sarcopenia patients. It is suggested that age has a slightly negative association with CAF levels, however this association is statistically insignificant (*p* = 0.44), as shown in Figure S6.

Subgroup analysis for the source of CAF concentration indicated that serum provided better results (ROM = 2.04 with 95% CI [1.50 to 2.59], *p* = 0.00) compared with plasma (ROM = 1.89 with 95% CI [1.32 to 2.45], *p* = 0.00) (Figure S3). The heterogeneity in the serum group was slightly reduced (*I* ^2^ = 89.59%), suggesting that serum samples might contribute to overall heterogeneity.

As shown in Figure S5, the subgroup analysis for the assessment criteria of sarcopenia revealed that there was no significant difference in either assessment criteria, that is, EWGSOP and EWGSOP2 with (ROM = 1.64 with 95% CI [1.36 to 1.91], *p* = 0.00) and (ROM = 1.60 with 95% CI [0.83 to 2.38], *p* = 0.00), respectively. However, in one study conducted by Xu et al. [49], sarcopenia was assessed by AWGS 2019 and that might be the source heterogeneity.

Because our main analysis included both primary and secondary sarcopenic individuals, we performed subgroup analysis for CAF concentration among these groups to see if it could explain the heterogeneity (Figure S4). However, heterogeneity remained high in both groups (i.e., *I* ^2^ = 98.08% and 98.57%, respectively). To further understand the source of heterogeneity, we also conducted leave‐one‐out analysis, but it did not clarify the source of heterogeneity (Tables S4–S6).

### 3.9 Association Between CAF Levels, Sarcopenia and Factors Associated With Sarcopenia

The relationship between CAF and sarcopenia is becoming increasingly evident in the literature. Numerous studies have established a consistent correlation between elevated levels of CAF and the presence of sarcopenia, underscoring the potential CAF as a biomarker for muscle health.

For instance, as shown in Table 1, Marzetti et al. [10] conducted a cross‐sectional study that focused on hip fracture patients. Their findings revealed that individuals diagnosed with sarcopenia had significantly higher levels of CAF compared with those without the condition. This suggests that elevated CAF levels may be a reliable indicator of muscle degradation in older adults, particularly in population prone to fractures, where muscle strength and mass are critical for recovery and overall mobility.

Further supporting this association, research involving patients with chronic conditions has shown similar trends. In studies assessing populations with chronic obstructive pulmonary disease (COPD) and diabetes, elevated CAF levels were consistently linked to decrease in muscle mass and strength. Karim et al. [47] reported findings in patients with Parkinson's disease, highlighting that higher CAF concentrations were associated with sarcopenic changes, reinforcing the idea that CAF levels reflect not only muscle condition but also the broader impact of chronic diseases on muscle health.

Moreover, studies by Pratt et al. [50] further elaborates on the association between CAF levels and sarcopenia. Their cohort analysis indicated that individuals with sarcopenia had significantly elevated CAF levels compared with their non‐sarcopenic individuals. The findings from these studies suggest a clear gradient where increased CAF levels correlate with both the presence and severity of sarcopenia. This relationship implies that CAF may serve not only as a marker for assessing muscle health but also as an indicator of the underlying inflammatory processes that contribute to muscle degeneration.

## 4 Discussion

Sarcopenia is primarily assessed by measuring muscle mass and strength, which requires valid measuring equipment and methods that are not readily available in local community settings. Therefore, although sarcopenia increases the risk of adverse health outcomes, its assessment in primary health care is still challenging [53]. To address this concern, researchers have investigated various biological markers in blood for accurate and early detection of sarcopenia, among which the biomarkers of NMJ, particularly the CAF, has recently gained prime importance [20]. Hence, this meta‐analysis and systematic review is based on the earlier investigations verifying the significant association of CAF with sarcopenia.

The main findings of this review further confirm the relationship between elevated blood CAF levels and sarcopenia. CAF levels in primary sarcopenic group were higher with an effects size of 1.93 (ROM = 1.93, 95% CI [1.49 to 2.36]; *p* = 0.00) than non‐sarcopenic group. Similarly, when secondary sarcopenic individuals were compared with non‐sarcopenic (having co‐morbidities other than sarcopenia) individuals, the CAF levels were consistently higher in secondary sarcopenic individuals with an effect size of 1.38 (ROM = 1.38, 95% CI [0.94 to 1.83], *p* = 0.00).

Additionally, to ensure our main findings, we also explored the relationship of CAF with key parameters of sarcopenia (HGS < 30 kg and < 20 kg while SMI as < 7 kg/m^2^ and < 5.5 kg/m^2^ in males and females, respectively) as defined by EWSGOP [52]. CAF levels were higher in individuals with low HGS and SMI with an effect size of 1.09 (ROM = 1.09 with 95% CI [1.05 to 1.13], *p* = 0.00) and 1.10 (ROM = 1.10 with 95% CI [1.05 to 1.14], *p* = 0.00), respectively, when compared with those having normal values. Furthermore, our meta‐regression analysis did not show statistically significant association between age and CAF levels, suggesting that while age is a common cause of sarcopenia, the higher CAF levels associated with sarcopenia are not directly related to age. These results highlight the importance of elevated CAF levels in the assessment of sarcopenia.

Moreover, our meta‐analysis showed substantial heterogeneity among studies, underscoring the complex nature of sarcopenia and suggesting that factors like gender of participants, different sarcopenia diagnosis criteria and age of participants may contribute to the observed differences in sarcopenic and non‐sarcopenic individuals. Despite this heterogeneity, the consistent finding of elevated CAF levels in sarcopenic individuals across studies suggests that CAF might be a potential biomarker for early diagnosis of sarcopenia.

Supporting the validity of our results, a recent study conducted by Landi et al. [45] showed that higher serum CAF levels were associated with sarcopenia and were not dependent on age, sex, physical, or other biological factors, suggesting CAF as a significant biomarker for screening sarcopenia in community dwellers. Pratt et al. [50] found a relationship between CAF and skeletal muscle mass in a large population, suggesting that CAF can be used as a reliable and cost‐effective diagnostic tool for sarcopenia. The pathogenesis of sarcopenia involves age‐related impairments at the NMJ, which compromises the interplay between nerve and muscle. CAF present in the bloodstream not only increases neurotrypsin activity but also decreases the strength of NMJ. Xu et al. [49] demonstrated that serum CAF concentration was higher in sarcopenic individuals compared with those without this condition. They also found elevated CAF levels were negatively related with HGS, especially in men, suggesting that patients with decreased HGS had higher CAF concentrations in the blood. Additionally, serum CAF levels have been associated with sarcopenia in multimorbid patients [49]. Marzetti et al. [10] showed that serum CAF levels were associated with sarcopenia in hip fracture patients, with higher levels in sarcopenic patients compared with non‐sarcopenic patients of the same age, indicating increased neurotrypsin activity and decreased NMJ strength due to cell damage caused by oxidative stress. Furthermore, in another study, it was expected that patients with lower endogenous antioxidant capacity (as in sarcopenia) would have higher CAF levels because the results showed elevated CAF levels in entire hip fracture patients, representing symptoms of NMJ degradation. However, during the assessment of sarcopenia according to Janssen's criteria, peripheral CAF concentration appeared to be higher in hip fractured sarcopenic patients when compared with hip fracture non‐sarcopenic patients [44].

While this meta‐analysis contributes to the growing evidence supporting CAF as an emerging biomarker for sarcopenia, several limitations must be considered. The significant heterogeneity suggests that unmeasured confounding factors like gender, age of study participants and different sarcopenia assessment tools might influence the relationship between CAF and sarcopenia. Moreover, most studies included in this meta‐analysis were cross‐sectional, limiting the ability to infer causality. Although higher CAF concentration is associated with sarcopenia, it remains unclear whether elevated CAF levels are a risk factor for sarcopenia or a consequence of the condition. Therefore, longitudinal studies are needed to elucidate the temporal relationship between CAF levels and sarcopenia progression.

In conclusion, this meta‐analysis supports the potential of CAF as a biomarker for sarcopenia, with elevated levels observed in sarcopenic individuals and those with decreased HGS and SMI. CAF could serve as a valuable tool for the early diagnosis and monitoring of sarcopenia, ultimately facilitating the management and treatment of this debilitating condition. Further studies are warranted to confirm these findings and to elucidate the biological pathways involved in the association between CAF and sarcopenia.

## Conflicts of Interest

The authors declare no conflicts of interest.

## Supporting information


**Table S1.** Quality assessment of the included studies using the JBI critical appraisal tool for case–control studies.
**Table S2.** Quality assessment of the included studies using the JBI critical appraisal tool for cohort studies.
**Table S3.** Quality assessment of the included studies using the JBI critical appraisal tool for cross‐sectional studies.
**Table S4.** Leave‐one‐out sensitivity analyses on CAF levels in sarcopenic and non‐sarcopenic individuals to investigate the source of heterogeneity.
**Table S5.** Leave‐one‐out sensitivity analyses on CAF levels in sarcopenic and non‐sarcopenic (individuals with co‐morbidities other than sarcopenia) individuals to investigate the source of heterogeneity.
**Table S6.** Leave‐one‐out sensitivity analyses on CAF levels in patients with low Grip strength to investigate the source of heterogeneity.
**Figure S1.** Funnel plot of the Ratio of Mean (ROM) for CAF levels in sarcopenic and non‐sarcopenic individuals, showing publication bias with pseudo 95% CI.
**Figure S2.** Forest plot of the Ratio of Mean (ROM) with 95% CI for CAF concentration among different age groups.
**Figure S3.** Forest plot of the Ratio of Mean (ROM) with 95% CI for CAF concentration obtained from different sources.
**Figure S4.** Forest plot of the Ratio of Mean (ROM) with 95% CI for CAF concentration among different sarcopenia groups.
**Figure S5.** Forest plot of the Ratio of Mean (ROM) with 95% CI for assessment criteria of sarcopenia.
**Figure S6.** Bubble plot with a fitted meta‐regression line of the correlation between Ratio of Mean (ROM) of CAF concentration and the age of patients with sarcopenia. Circles are sized according to the precision of each estimate (the inverse of its within‐study variance).

## Data Availability

All data generated or analysed during this study are included in this published article.
