# Supplementary material for: C‐Terminal Agrin Fragment as a Biomarker for Sarcopenia: A Systematic Review and Meta‐Analysis
Source: J Cachexia Sarcopenia Muscle. 2025 Jan 30;16(1):e13707. doi: 10.1002/jcsm.13707 (PMC11780277; doi:10.1002/jcsm.13707)

**Supplementary Data:**

**Contents:**

**1. Supplementary Tables:**

**Supplementary Table 1.** Quality assessment of the included studies using the JBI critical appraisal tool for case-control studies.

**Supplementary Table 2.** Quality assessment of the included studies using the JBI critical appraisal tool for cohort studies.

**Supplementary Table 3.** Quality assessment of the included studies using the JBI critical appraisal tool for cross-sectional studies.

**Supplementary Table 4.** Leave-one-out sensitivity analyses on CAF levels in sarcopenic and non-sarcopenic individuals to investigate the source of heterogeneity.

**Supplementary Table 5.** Leave-one-out sensitivity analyses on CAF levels in sarcopenic and non-sarcopenic (individuals with co-morbidities other than sarcopenia) individuals to investigate the source of heterogeneity.

**Supplementary Table 6.** Leave-one-out sensitivity analyses on CAF levels in patients with low Grip strength to investigate the source of heterogeneity.

**2. Supplementary Figures:**

**Supplementary Figure 1.** Funnel plot of the Ratio of Mean (ROM) for CAF levels in sarcopenic and non-sarcopenic individuals, showing publication bias with pseudo 95% CI.

**Supplementary Figure 2**. Forest plot of the Ratio of Mean (ROM) with 95% CI for CAF concentration among different age groups.

**Supplementary Figure 3**. Forest plot of the Ratio of Mean (ROM) with 95% CI for CAF concentration obtained from different sources.

**Supplementary Figure 4**. Forest plot of the Ratio of Mean (ROM) with 95% CI for CAF concentration among different sarcopenia groups.

**Supplementary Figure 5**. Forest plot of the Ratio of Mean (ROM) with 95% CI for assessment criteria of sarcopenia.

**Supplementary Figure 6**. Bubble plot with a fitted meta-regression line of the correlation between Ratio of Mean (ROM) of CAF concentration and the age of patients with sarcopenia. Circles are sized according to the precision of each estimate (the inverse of its within-study variance).

**Supplementary Table 1.** Quality assessment of the included studies using the JBI critical appraisal tool for case-control studies.

| **Quality scores (from 1-10)** | | | | | | | | | | | | | |
| --- | --- | --- | --- | --- | --- | --- | --- | --- | --- | --- | --- | --- | --- |
| **Items** | | | | | | | | | | | | | **Quality scores (from 1-10; higher scores indicate less risk of bias)** |
| **Study** | **Study Design** | **1** | **2** | **3** | **4** | **5** | **6** | **7** | **8** | **9** | **10** | **Overall appraisal** | **Total** |
| Hettwer et. al, 2013 | Case-control | Y | Y | Y | Y | Y | N | N | Y | Y | Y | I | 8 |
| Al Rubaye et. al, 2020 | Case-control | Y | Y | Y | Y | Y | N | N | Y | Y | Y | I | 8 |

**Legends:** Item.1- Were the groups comparable other than the presence of disease in cases or the absence of disease in controls? Item. 2 - Were cases and controls matched appropriately? Item.3 - Were the same criteria used for the identification of cases and controls? Item.4 - Was exposure measured in a standard, valid and reliable way? Item.5 - Was exposure measured in the same way for cases and controls? Item.6 - Were confounding factors identified? Item.7 - Were strategies to deal with confounding factors stated? Item. 8- Were outcomes assessed in a standard, valid and reliable way for cases and controls? Item.9 - Was the exposure period of interest long enough to be meaningful? Item.10 - Was appropriate statistical analysis used?

**Abbreviations:** Y, yes; N, no; U, Unclear; N/A, not applicable; I, Included

| **Quality scores (from 1-10)** | | | | | | | | | | | | | |  |
| --- | --- | --- | --- | --- | --- | --- | --- | --- | --- | --- | --- | --- | --- | --- |
| **Items** | | | | | | | | | | | | | | **Quality scores (from 1-11; higher scores indicate less risk of bias)** |
| **Study** | **Study Design** | **1** | **2** | **3** | **4** | **5** | **6** | **7** | **8** | **9** | **10** | **11** | **Overall Appraisal** | **Total** |
| Pratt et. al, 2021 | Cohort | Y | Y | Y | N | N | Y | Y | Y | Y | N/A | Y | I | 8 |
| Karim et. al, 2022 (a) | Cohort | Y | Y | Y | N | N | Y | Y | Y | Y | N/A | Y | I | 8 |
| Asima Karim et. al, 2022 | Cohort | Y | Y | Y | N | N | Y | Y | Y | Y | N/A | Y | I | 8 |
| Karim et. al, 2021 | Cohort | Y | Y | Y | N | N | Y | Y | Y | Y | N/A | Y | I | 8 |
| Karim et. al, 2022 (b) | Cohort | Y | Y | Y | N | N | Y | Y | Y | Y | N/A | Y | I | 8 |
| Landi et. al, 2016 | Cohort | Y | Y | Y | N | N | Y | Y | Y | Y | N/A | Y | I | 8 |

**Supplementary Table 2.** Quality assessment of the included studies using the JBI critical appraisal tool for cohort studies.

**Legends:** Item.1- Were the two groups similar and recruited from the same population? Item. 2 - Were the exposures measured similarly to assign people to both exposed and unexposed groups? Item.3 - Was the exposure measured validly and reliably? Item.4 - Were confounding factors identified? Item.5 - Were strategies to deal with confounding factors stated? Item.6 - Were the groups/participants free of the outcome at the start of the study (or at the moment of exposure)? Item.7 - Were the outcomes measured validly and reliably? Item. 8- Was the follow-up time reported and sufficient to be long enough for outcomes to occur? Item.9 Was follow-up complete, and if not, were the loss reasons to follow up described and explored? Item.10 - Were strategies to address incomplete follow-up utilized? Item.11 - Was appropriate statistical analysis used?

**Abbreviations:** Y, yes; N, no; U, Unclear; N/A, not applicable; I, Included.

**Supplementary Table 3.** Quality assessment of the included studies using the JBI critical appraisal tool for cross-sectional studies.

| **Quality scores (from 1-10)** | | | | | | | | | | | |
| --- | --- | --- | --- | --- | --- | --- | --- | --- | --- | --- | --- |
| **Items** | | | | | | | | | | | **Quality scores (from 1-10; higher scores indicate less risk of bias)** |
| **Study** | **Study Design** | **1** | **2** | **3** | **4** | **5** | **6** | **7** | **8** | **Overall appraisal** | **Total** |
| Pratt et. al, 2024 | Cross-sectional | Y | Y | Y | Y | N | N | Y | Y | I | 6 |
| Qun Xu et. al, 2023 | Cross-sectional | Y | Y | Y | Y | N | N | Y | Y | I | 6 |
| Marzetti et. al, 2014 | Cross-sectional | Y | Y | Y | Y | N | N | Y | Y | I | 6 |

**Legends:** Item.1- Were the criteria for inclusion in the sample clearly defined? Item. 2 – Were the study subjects and the setting described in detail? Item.3 – Was the exposure measured in a valid and reliable way? Item.4 – Were objectives, standard criteria used for measurement of the condition? Item.5 - Was confounding factors identified? Item.6 – Were strategies to deal with confounding factors stated? Item.7 - Were the outcomes measured in a valid and reliable way? Item. 8- Was appropriate statistical analysis used?

**Abbreviations:** Y, yes; N, no; U, Unclear; N/A, not applicable; I, Included.

**Supplementary Table 4:** Leave-one-out sensitivity analyses on CAF levels in sarcopenic and non-sarcopenic individuals to investigate the source of heterogeneity.

|  | **ROM with 95% CI** | **Between-study heterogeneity** |
| --- | --- | --- |
| **Overall** | 1.93 [1.49, 2.36] | *p* = 0.00; I ^2^ = 98.82% |
| **Omitted study** | | |
| Pratt et. al, 2021 | 2.03 [1.60, 2.47] | *p* = 0.00; I^2^ = 98.71% |
| Hettwer et. al, 2012 | 1.95 [1.45, 2.44] | *p* = 0.00; I^2^ = 99.01% |
| Pratt et. al, 2024 | 2.03 [1.58, 2.47] | *p* = 0.00; I^2^ = 98.28% |
| Al Rubaye et. al, 2020 | 1.87 [1.39, 2.35] | *p* = 0.00; I^2^ = 99.04% |
| Karim et. al, 2022 (a) | 1.86 [1.38, 2.33] | *p* = 0.00; I^2^ = 98.92% |
| Asima Karim et. al, 2022 | 1.94 [1.44, 2.44] | *p* = 0.00; I^2^ = 99.08% |
| Karim et. al, 2021 | 1.97 [1.48, 2.46] | *p* = 0.00; I^2^ = 98.77% |
| Karim et. al, 2022 (b) | 1.77 [1.41, 2.12] | *p* = 0.00; I^2^ = 97.93% |

**Supplementary Table 5:** Leave-one-out sensitivity analyses on CAF levels in sarcopenic and non-sarcopenic (individuals with co-morbidities other than sarcopenia) individuals to investigate the source of heterogeneity.

|  | **ROM with 95% CI** | **Between-study heterogeneity** |
| --- | --- | --- |
| **Overall** | 1.38 [0.94, 1.83] | *p* = 0.00; I^2^ = 98.38% |
| **Omitted study** | | |
| Qun Xu et. al, 2023 | 1.54 [0.97, 2.12] | *p* = 0.00; I^2^ = 94.97% |
| Marzetti et. al, 2014 | 1.18 [0.98, 1.37] | *p* = 0.00; I^2^ = 92.72% |
| Landi et. al, 2016 | 1.45 [0.68, 2.22] | *p* = 0.00; I^2^ = 96.74% |

**Supplementary Table 6:** Leave-one-out sensitivity analyses on CAF levels in patients with low Grip strength to investigate the source of heterogeneity.

|  | **ROM with 95% CI** | **Between-study heterogeneity** |
| --- | --- | --- |
| **Overall** | 1.09 [1.05, 1.13] | *p* = 0.00; I^2^ = 0.01% |
| **Omitted study** | | |
| Pratt et. al, 2021 | 1.09 [1.04, 1.24] | *p* = 0.00; I^2^ = 19.24% |
| Qun Xu et. al, 2023 | 1.08 [1.03, 1.13] | *p* = 0.00; I^2^ = 0.01% |
| Pratt et. al, 2024 | 1.12 [1.05, 1.19] | *p* = 0.00; I^2^ = 0.03% |

**Supplementary Figure 1:** Funnel plot of the Ratio of Mean (ROM) for CAF levels in sarcopenic and non-sarcopenic individuals, showing publication bias with pseudo 95% CI.

**
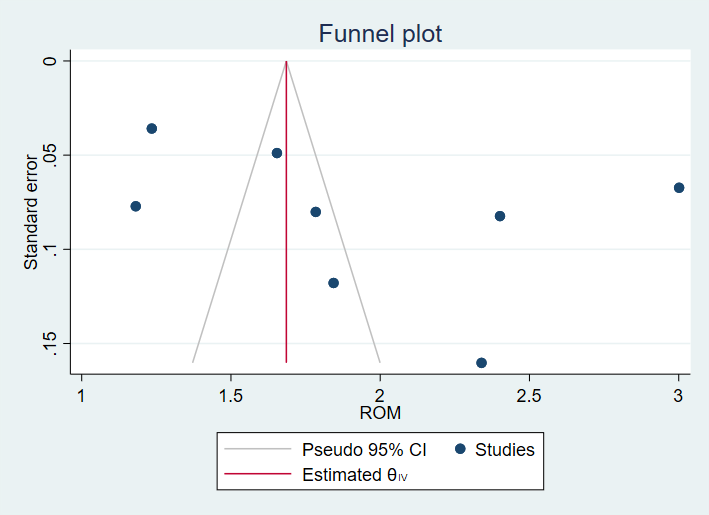
**

**Regression-based Egger test for small-study effects**

Random-effects model

Method: REML

Moderators: ROM and SEPool

H0: beta1 = 0; no small-study effects

beta1 = 5.43

SE of beta1 = 6.130

z = 0.89

Prob > |z| = 0.3760

**Supplementary Figure 2**: Forest plot of the Ratio of Mean (ROM) with 95% CI for CAF concentration among different age groups.


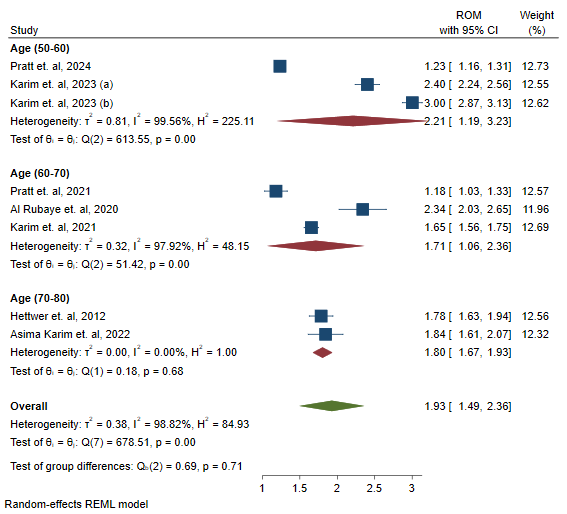


**Supplementary Figure 3**: Forest plot of the Ratio of Mean (ROM) with 95% CI for CAF concentration obtained from different sources.


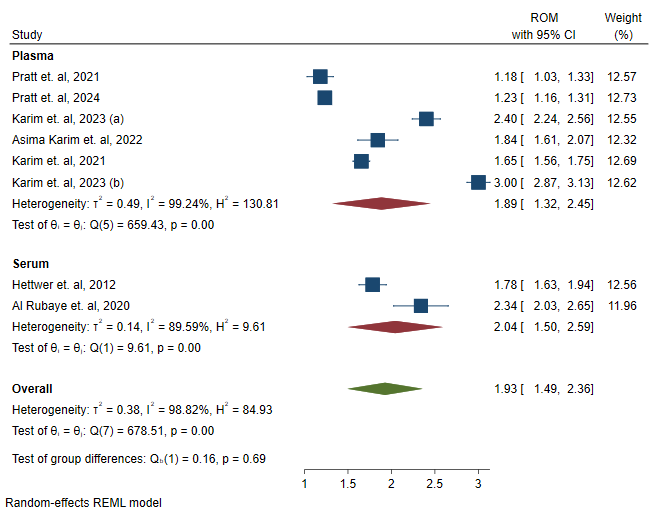


**Supplementary Figure 4**: Forest plot of the Ratio of Mean (ROM) with 95% CI for CAF concentration among different sarcopenia groups.


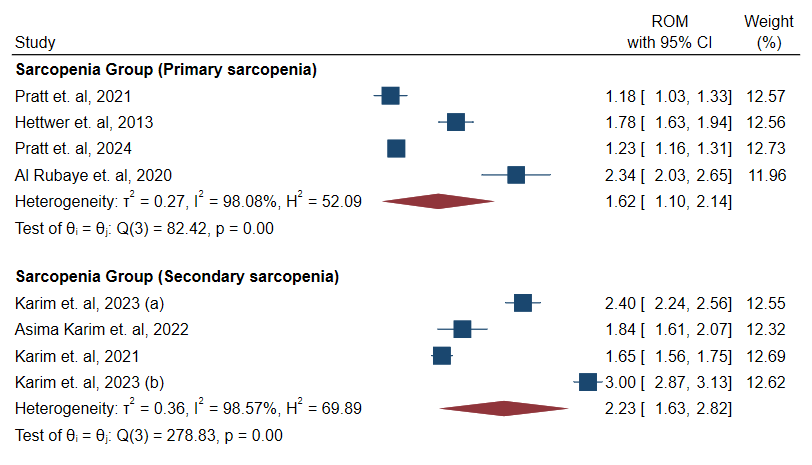


**Supplementary Figure 5**: Forest plot of the Ratio of Mean (ROM) with 95% CI for assessment criteria of sarcopenia.


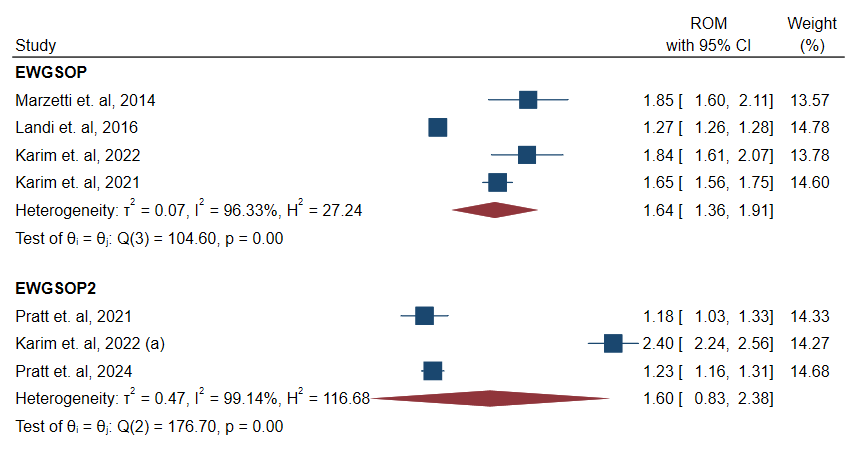


**Supplementary Figure 6**: Bubble plot with a fitted meta-regression line of the correlation between Ratio of Mean (ROM) of CAF concentration and the age of patients with sarcopenia. Circles are sized according to the precision of each estimate (the inverse of its within-study variance).


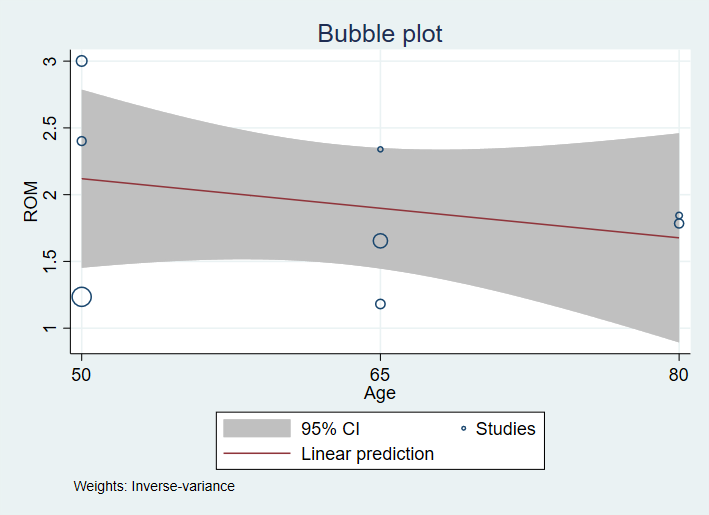

Supplement: Supplementary file 1 — Table S1. Quality assessment of the included studies using the JBI critical appraisal tool for case–control studies. Table S2. Quality assessment of the included studies using the JBI critical appraisal tool for cohort studies. Table S3. Quality assessment of the included studies using the JBI critical appraisal tool for cross‐sectional studies. Table S4. Leave‐one‐out sensitivity analyses on CAF levels in sarcopenic and non‐sarcopenic individuals to investigate the source of heterogeneity. Table S5. Leave‐one‐out sensitivity analyses on CAF levels in sarcopenic and non‐sarcopenic (individuals with co‐morbidities other than sarcopenia) individuals to investigate the source of heterogeneity. Table S6. Leave‐one‐out sensitivity analyses on CAF levels in patients with low Grip strength to investigate the source of heterogeneity. Figure S1. Funnel plot of the Ratio of Mean (ROM) for CAF levels in sarcopenic and non‐sarcopenic individuals, showing publication bias with pseudo 95% CI. Figure S2. Forest plot of the Ratio of Mean (ROM) with 95% CI for CAF concentration among different age groups. Figure S3. Forest plot of the Ratio of Mean (ROM) with 95% CI for CAF concentration obtained from different sources. Figure S4. Forest plot of the Ratio of Mean (ROM) with 95% CI for CAF concentration among different sarcopenia groups. Figure S5. Forest plot of the Ratio of Mean (ROM) with 95% CI for assessment criteria of sarcopenia. Figure S6. Bubble plot with a fitted meta‐regression line of the correlation between Ratio of Mean (ROM) of CAF concentration and the age of patients with sarcopenia. Circles are sized according to the precision of each estimate (the inverse of its within‐study variance). [file JCSM-16-e13707-s001.docx]
